# Metabolomics and Lipidomics Analyses Aid Model Classification of Type 2 Diabetes in Non-Human Primates

**DOI:** 10.3390/metabo14030159

**Published:** 2024-03-09

**Authors:** Peining Tao, Stacey Conarello, Thomas P. Wyche, Nanyan Rena Zhang, Keefe Chng, John Kang, Theodore R. Sana

**Affiliations:** 1 BARDS-Biometrics Research, Merck & Co., Inc., Rahway, NJ 07065, USA; jia.kang@merck.com; 2 QA Animal Welfare, Merck & Co., Inc., West Point, PA 19486, USA; stacey.conarello@merck.com; 3 Quantitative Biosciences, Merck & Co., Inc., Cambridge, MA 02141, USA; thomas.wyche@merck.com; 4 Pharmacokinetics, Dynamics, Metabolism & Bioanalytics, Merck & Co., Inc., West Point, PA 19486, USA; rena_zhang@merck.com; 5 Crown Bioscience Inc., San Diego, CA 92127, USA; keefechng@crownbio.com

**Keywords:** type 2 diabetes (T2D), metabolomics, plasma, feces, dysmetabolic and diabetic, non-human primates (NHPs), LC/MS, lipids, triglycerides, phosphatidylcholine, bile acids, latent multivariate modeling, OPLS-DA, random forest, machine learning, LOD, mTIC

## Abstract

Type 2 diabetes (T2D) is a global public health issue characterized by excess weight, abdominal obesity, dyslipidemia, hyperglycemia, and a progressive increase in insulin resistance. Human population studies of T2D development and its effects on systemic metabolism are confounded by many factors that cannot be controlled, complicating the interpretation of results and the identification of early biomarkers. Aged, sedentary, and overweight/obese non-human primates (NHPs) are one of the best animal models to mimic spontaneous T2D development in humans. We sought to identify and distinguish a set of plasma and/or fecal metabolite biomarkers, that have earlier disease onset predictability, and that could be evaluated for their predictability in subsequent T2D studies in human cohorts. In this study, a single plasma and fecal sample was collected from each animal in a colony of 57 healthy and dysmetabolic NHPs and analyzed for metabolomics and lipidomics. The samples were comprehensively analyzed using untargeted and targeted LC/MS/MS. The changes in each animal’s disease phenotype were monitored using IVGTT, HbA1c, and other clinical metrics, and correlated with their metabolic profile. The plasma and fecal lipids, as well as bile acid profiles, from Healthy, Dysmetabolic (Dys), and Diabetic (Dia) animals were compared. Following univariate and multivariate analyses, including adjustments for weight, age, and sex, several plasma lipid species were identified to be significantly different between these animal groups. Medium and long-chain plasma phosphatidylcholines (PCs) ranked highest at distinguishing Healthy from Dys animals, whereas plasma triglycerides (TG) primarily distinguished Dia from Dys animals. Random Forest (RF) analysis of fecal bile acids showed a reduction in the secondary bile acid glycoconjugate, GCDCA, in diseased animals (AUC 0.76[0.64, 0.89]). Moreover, metagenomics results revealed several bacterial species, belonging to the genera *Roseburia*, *Ruminococcus*, *Clostridium*, and *Streptococcus*, to be both significantly enriched in non-healthy animals and associated with secondary bile acid levels. In summary, our results highlight the detection of several elevated circulating plasma PCs and microbial species associated with fecal secondary bile acids in NHP dysmetabolic states. The lipids and metabolites we have identified may help researchers to differentiate individual NHPs more precisely between dysmetabolic and overtly diabetic states. This could help assign animals to study groups that are more likely to respond to potential therapies where a difference in efficacy might be anticipated between early vs. advanced disease.

## 1. Introduction

Type 2 diabetes (T2D) is a chronic metabolic disease, characterized by progressive loss of insulin secretion and hyperglycemia, ultimately resulting in adverse clinical complications. T2D has become a major global health problem: a consequence of high-calorie diets and sedentary lifestyles, factors that contribute to escalating healthcare costs. The World Health Organization (WHO) estimates that there are approximately 422 million people across the globe with diabetes, and 1.5 million deaths each year directly attributed to this disease. Moreover, the prevalence of prediabetes—a dysmetabolic condition with a high risk of progression to T2D—has been steadily increasing over the past two decades and is approximately two to three times higher than that of diabetes, with a yearly conversion rate of 5–10% [1]. Identification of early onset plasma or feces biomarkers and the development of more sensitive, pre-clinical risk prediction models in NHPs can potentially be very helpful in their translatability and application to studies of human cohorts. Dysregulated fatty acid metabolism and tissue lipid accumulation is associated with the development of insulin resistance and T2D [2,3,4]. However, there is often a latency between excessive hepatic fat accumulation and clinical symptom manifestations, which is associated with disease pathogenesis. Current stratification metrics for individuals at risk of developing T2D in the general population are based on well-established factors such as age, BMI, fasting glucose, blood pressure, family history, and glycated hemoglobin (HbA1c) levels [5]. The most typical lipid-based risk factors for assessing T2D development are HDL, LDL, and TG levels. Elevated circulating branched chain amino acid (BCAA) concentrations are well established as being correlated with human obesity and diabetic animal models of the disease [6,7,8,9]. A meta-analysis of metabolomics studies that identified metabolites associated with pre-diabetes and T2D highlighted several lipid classes: triglycerides, phospholipids, and sphingomyelins, as well as aromatic amino acids. Glycine and glutamine are inversely associated with the risk of T2D [10]. However, interpretation of their relative predictive value depends on being able to account for many confounding environmental factors, which are frequently challenging to overcome in studies of human cohorts.

NHPs are a particularly suitable animal model for studying disorders like obesity and diabetes [11] and are considered the gold standard for research into the study of the pathophysiology of obesity and diabetes [12,13]. 

NHPs present fewer confounding variables as they are housed in a controlled environment, have regular feeding times and feces collection schedules, and their body weight and food and liquid consumption are routinely monitored. Like in humans, T2D is most common in older, sedentary, obese NHPs. They share some of the same metabolic disease characteristics as humans: development of progressive changes in obesity, insulin resistance, and dyslipidemia [14]. 

The trajectory from an early dysmetabolic (i.e., pre-diabetic) state to overt T2D can often be managed pharmacologically, and/or through lifestyle changes such as diet and exercise. Hence, sensitive, prognostic metabolomic markers and modeling tools for NHPs would be useful for earlier classification of potentially at-risk animals, and for detecting early metabolite changes that could also positively impact disease management, resulting in fewer co-morbidities. In addition to a strong genetic association between obesity and T2D, lifestyle, nutrition, and the host’s microbiome can all have critical roles in disease development. 

This cohort of animals was followed for many years and routinely evaluated every 3–6 months for their metabolic state based on clinical chemistries, IVGTT, and body weight. Once we were certain of the metabolic state of each animal, one-time plasma and feces samples were collected. The aim of this pre-clinical study was to assess whether we could accurately categorize individual NHPs, based on their metabolite/lipid profiles, into pre-determined metabolic states. If successful, future metabolomics and metagenomics studies of plasma and feces samples could be particularly valuable for regularly monitoring NHP progression between healthy and dysmetabolic to overtly diabetic states. Moreover, the identification of prognostic biomarkers in these matrixes could reveal important information about the efficacy of new treatments. 

Therefore, our objectives were to first test and adjust for confounding factors; to determine whether one-time collection and analysis of plasma and feces provided a relatively simple way to accurately categorize the animals based on metabolite and lipid profiles; to assess differences in disease classification between plasma and feces, and calculate which metabolites/lipids had the highest correlation with HbA1c; lastly, we evaluated whether metabolomics and metagenomics analyses of feces samples were associated with T2D. 

We analyzed the ultra-high-performance liquid chromatography–tandem mass spectrometry (UHPLC MS/MS) results of plasma and fecal sample extracts from 57 NHPs: 17 Healthy, 16 Dys, and 24 Dia. The most important metabolites for classifying each disease state were identified after rigorous data pre-processing; adjustment for confounders; the application of multiplicity correction to compensate for the error rate of post-hoc data analysis; machine learning; and metabolite chemical similarity enrichment analyses [15]. Furthermore, for a subset of animals, we performed metagenomics analyses of their fecal microbiome samples to reveal whether the abundance of distinct bacterial taxonomic groups were positively associated with fecal metabolite data (such as bile acids), and supported the animals’ disease status classification. 

## 2. Materials and Methods

### 2.1. Animal Housing and Husbandry

All animals (16 Dys, 24 Dia, and 17 Healthy) were housed at Crown Bioscience Louisiana (New Iberia, LA, USA), in individual Group 5 stainless steel wire-bottomed cages (as defined by the Guide for the Care and Use of Laboratory Animals of the Institute for Laboratory Animal Research/National Academies of Science), in rooms with an ambient temperature of 22–26 °C, a relative humidity of 40–70%, and a 12 h light–dark cycle. Samples were collected in accordance with the “Institutional Animal Care and Use Committee-approved standard operating procedures”. Animals were fed ad libitum twice a day on Purina LabDiet—5037, Jumbo Monkey Chow. In addition to standard maintenance (not high fat, high cholesterol, or high fructose) pelleted chow, fresh fruit was provided daily, and State-supplied water was always freely available.

### 2.2. One-Time Plasma and Feces Collection

This colony was monitored as part of a long-term observational NHP study—“Animal Holding and Phenotyping of Dysmetabolic State in Rhesus Macaques”—and the animals were routinely (and continually) evaluated for their metabolic state based on clinical chemistries. Given the chronicity, repeated measures, and laboratory test over time, we were confident of the accuracy of their diabetic status. There was a limited time window to collect one-time blood and fecal samples from each animal in this colony for metabolomics/lipidomics analysis. Due to their different collection schedules, the samples were collected over a two-month period. The date, time, and metabolic state of each animal was documented during sample collection. The animals were fasted overnight and sedated with Ketamine prior to whole blood collection into K_2_EDTA tubes (BD^TM^ P800), which was then centrifuged and the plasma was transferred into 1.5 mL Eppendorf tubes, frozen, and stored at −20 °C prior to shipment for analysis.

For feces collection, the NHPs were not fasted. In the morning, feces samples were passed by the animals onto clean trays containing absorbent pads. Approximately 3–5 g of solid feces sample were transferred into sterile 50 mL Falcon tubes with sterile forceps and were then immediately frozen and stored at −80 °C before shipment for analysis.

### 2.3. Animal Phenotype Characterization

All animals were enrolled in the institutional environmental enrichment program and monitored throughout the study in consultation with the institution’s clinical veterinarian for any signs of change in their physiological and/or psychological state, as well as changes to their physical appearance and health, e.g., body weight, hair coat quality, loss of appetite, and gastrointestinal changes.

The NHP colony was monitored every 3 to 6 months to assess their phenotype/metabolic state using several parameters, including intravenous glucose tolerance tests (IVGTT), body weight, serum chemistries, and hematology profiles. In healthy animals, typically two spikes in insulin levels were observed: an insulin mobilization spike, followed by an insulin de-granularization spike [16]. In healthy animals the mean HbA1C levels are not expected to change significantly with aging [17]. As NHPs become diabetic, however, a second insulin spike gradually disappears. The colony was monitored over time for progression from a Healthy to Dys/Dia disease state, and grouped into one of three metabolic states, based on the following HbA1c ranges [18]: Healthy (HbA1c < 4.5%); dysmetabolic (Dys) (HbA1c 4.5–6%); and diabetic (Dia) (HbA1c > 6%).

### 2.4. Sample Processing and LC-MS Data Acquisition

Sample processing and data acquisition details for NHP plasma and fecal samples are available from the NIH West Coast Metabolomics Center (WCMC) and can be accessed under each specific “Data Dictionary Fiehn laboratory NIH West Coast Metabolomics Center” file (see Supplemental files for each panel). The metabolite panels were analyzed using validated chromatography/mass spectrometry methods as described on the West Coast Metabolomic Center (WCMC) Core facility website. Briefly, untargeted biogenic amine analysis through HILIC-QTOF MS/MS was performed on plasma extracts with a SCIEX TripleTOF^®^ 6600 Quadrupole Time-Of-Flight (QTOF) mass analyzer. Untargeted complex lipid analysis (CSH-QTOF MS/MS) was performed with an Agilent 6530B Accurate Mass Q-TOF LC/MS System. Lipids were detected by ESI(+) and/or ESI(−) combined. Targeted analyses of bile acids in plasma and feces were performed using a Waters UPLC/AB SCIEX QTRAP 6500 with targeted MRM panels.

### 2.5. Metabolite Panels

#### 2.5.1. Targeted Panels

For plasma, the AbsoluteIDQ^®^ p180 kit (Biocrates Life Sciences AG, Innsbruck, Austria) was used for targeted, quantitative analysis of metabolites, according to the manufacturer’s instructions.

A targeted bile acid panel was processed and analyzed at the WCMC. The panel included: Taurodehydrocholate, Tauro-ω-Muricholic acid, Tauro-α-Muricholic acid, Tauro-β-Muricholic acid, Tauroursodeoxycholic acid, Taurocholic acid, ω-Muricholic acid, Glycoursodeoxycholic acid, Glycohyodeoxycholic acid, α-Muricholic acid, Glycocholic acid, β-Muricholic acid, Taurochenodeoxycholic acid, Taurodeoxycholic acid, Cholic acid, Ursodeoxycholic acid, Glycochenodeoxycholic acid, Glycodeoxycholic acid, Taurolithocholic acid, Chenodeoxycholic acid, Deoxycholic acid, Glycolithocholic acid, and Lithocholic acid.

The LLOQ (lower limit of quantitation) and ULOQ (upper limit of quantitation) for each compound was obtained from WCMC. For each metabolite, measurements below the LLOQ were imputed as one half of its LLOQ. The post-imputed concentration data was subsequently scaled and log-transformed prior to downstream statistical analyses.

#### 2.5.2. Untargeted Metabolite Panels

A plasma lipids panel was analyzed at the WCMC using CSH-QTOF MS/MS. Normalized relative intensities were reported for unknown structures, and a total of 394 lipids were annotated that had been identified by accurate mass, RT, and matching the WCMC’s mass spectral library of approximately 400,000 MS/MS spectra, using their LipidBlast database [19]. Our plasma lipid panel included 394 lipids that were divided into several lipid classes, including (see Supplementary Table S1) 93 triglycerides (TG), 127 phosphatidylcholines (PC), 30 sphingomyelins (SM), 23 lysophosphatidylcholines (LPC), 29 fatty acids (FA), 27 phosphatidylethanolamines (PE), 25 ceramides (Cer), 12 diacylglycerols (DG), 10 cholesterol esters (CE), 10 acylcarnitines (AC), 4 lysophosphatidylethanolamines (LPE), 3 glucosylceramides (GlcCer), and cholesterol. The BinBase [20] database (a large open-access reference library of metabolite annotations based on LC-MS/MS retention times and mass spectral data) was used for matching to untargeted, HILIC-based LC-QTOF-MS results.

### 2.6. Untargeted and Targeted Data Processing

We determined the assay performance for each platform based upon the repeatability of several pooled QC samples (Figure S1, Supplementary). Untargeted lipidomics and HILIC-based LC-QTOFMS data were processed using MS-DIAL 2.84 software [21].

Data processing, filtering, normalization, and untargeted data transformation steps prior to identification and statistical analysis are summarized below:

- Filtered contaminating peaks in blank samples from biological data files.
- Excluded extreme outlier technical replicates in QC samples.
- Calculated the RSD% of QC samples, and flagged metabolites showing RSD > 30%
- Measurements below detection were imputed with one half of the lowest observed peak intensity.
- Batch/injection order was corrected using QC samples.
- Data were normalized using the sum of the known metabolites or “mTIC”
- Post-normalized peak intensities were scaled and/or log transformed.

### 2.7. Metabolomics and Lipidomics Statistics Analysis and Modeling

Statistical analyses were performed on a post hoc basis. Potential confounder variables such as age, body weight (BW), and sex [22] were adjusted prior to identifying differentially expressed metabolites.

Our plasma lipid panel was divided into 13 lipid classes, including 93 TGs, 127 PCs, and 30 SMs (see Supplementary Table S1). We initially used detrending [23] to diminish the influence of sex, age, and body weight on plasma lipid data. We then applied both multivariate Partial Least Squares Discriminant Analysis (OPLS-DA) [24,25] and a univariate Student’s t-test to distinguish between plasma lipids associated with different animal group phenotypes: Healthy, Dys, and Dia. OPLS-DA is particularly useful with high-dimensional biomarker data, such as lipidomics, that possess significant biomarker correlation. It was therefore particularly useful for analyzing our lipid data. Lipids were subsequently ranked by their Variable Influence in Projection (VIP) score [26], where a higher VIP score indicates a greater influence on discriminating phenotypes. Univariate two-sample t-tests were conducted to obtain the statistical significance for each lipid, followed by multiplicity correction. Hence, a list of significantly different lipids that discriminated between Healthy, Dys, and Dia animal groups was determined by a combination of univariate and multivariate approaches [27,28]. We filtered the list of lipids further by applying FDR < 0.05, fold change > 1.5, and a VIP score of >1. We subsequently used Generally Applicable Gene-set Enrichment (GAGE) analysis [29], using the VIP score as input to select lipid classes that best differentiated between Healthy, Dys, and Dia animals.

For fecal bile acid data, we utilized propensity score matching (PSM) [27] to balance sex, age, and body weight, assigning each NHP sample a propensity score. With the propensity score as input, we applied multivariate analysis using Weighted Random Forest. We applied univariate analysis with a weighted t-test for classification of the animal groups. Metabolites were ranked by their variable importance (VIMP) score, generated by the RF algorithm, where a higher VIMP score reflected greater importance in discriminating between animal groups. Univariate analysis was performed to determine the statistical significance for each metabolite. A combination of both the VIMP and the statistical significance was subsequently used to select metabolites that best differentiated between Healthy, Dys, and Dia animal groups.

### 2.8. Metagenomics Analysis

For consistency with some of the bile acid metabolomics data, the Dys/Dia fecal samples were pooled for metagenomics analysis. Feces samples were sequenced at Diversigen (New Brighton, MN, USA), a CRO that performs metagenomics analyses and comprehensive genomic profiling of microbial populations using high-coverage shotgun DNA sequencing, on the Illumina HiSeq™ Sequencing System (San Diego, CA, USA). DNA extraction was performed with the QIAGEN (Hilden, Germany) DNeasy^®^ PowerSoil Kit^®^. The fastq files received from Diversigen were subsequently processed using an internal pipeline including adapter trimming; removal of PhiX control and human contaminating reads; and metagenomic taxonomic profiling. The MUSiCC metagenomic framework [30] for the normalization and accurate profiling of microbial gene abundances was applied to the taxonomy data prior to the downstream association analyses with T2D disease status. All statistical analyses were performed in the R-language.

## 3. Results

### 3.1. Clinical and Demographic Adjustments for Animal Confounding Factors

The rationale for applying detrending to our plasma data was backed up by the literature. Plasma TG levels and risks of metabolic disease are influenced by age [31]. There is also a significant correlation between plasma TG and obesity [32]. Since our results showed a borderline significant correlation between TG and age, and between TG and body weight (BW), detrending effectively reduced these influences prior to classification of the animals’ phenotypes (Healthy, Dys, and Dia) based on their lipid profiles. Moreover, subsequent OPLS did not incorporate weighted scores from PSM, so after detrending we only applied OPLS to lipidomics data. As for the fecal bile acid data, the correlation of body weight, age, and sex with fecal bile acids is not very clear. PSM was appropriate for rectifying confounding factors and was used in conjunction with Weighted Random Forest.

A summary of the demographic variables for the three animal groups, the number of animals per group, as well as some of the demographic and key clinical variables, including HbA1c levels, are summarized in Table 1. Of the 57 animals, when categorized by sex, the mean TG levels across all three groups of animals were 301.43 mg/dl for females (total of 16 animals) and 176.41 mg/dl for males (total of 41 animals). When both female and male animals were grouped together by phenotype, the mean TG levels were 360.5 mg/dL (Dia, 24 animals); 157.1 mg/dl (Dys, 16 animals); and 62.0 mg/dL (Healthy, 17 animals). Since age, sex, and body weight were not balanced between the three animal groups, statistical strategies were applied to adjust for these confounding factors.

Our results showed a borderline significant correlation (Spearman) coefficient of 0.26 (*p* = 0.05) between TG levels and age, and 0.23 (*p* = 0.1) between TG levels and body weight (BW). We therefore employed detrending to minimize the influence of sex, age, and body weight on the plasma lipid data and propensity score matching (PSM) to adjust for the fecal bile acid data.

### 3.2. Plasma Lipids and Fecal Metabolites Independently Aid in Animal Disease Phenotype Classification

**Figure 1:**
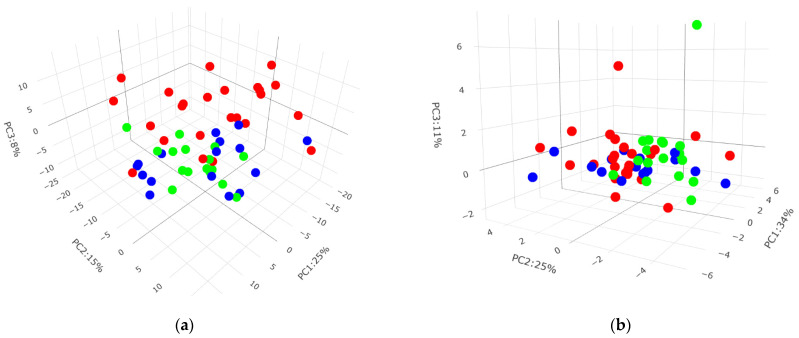
PCA plots of (**a**) plasma lipids and (**b**) fecal bile acids. Healthy (green), Dys (blue), and Dia (red) solid dots.

Prior to more in-depth data analyses, unsupervised PCA analysis of high-dimensional data served as the first step in detecting variance between the different animal groups. Separate PCA plots were generated for complex lipids in Figure 1a and bile acids in Figure 1b, the two main classes of metabolites for plasma and feces, respectively. The PCA plots were rotated in the direction where best separation could be observed. The plasma lipids’ PCA results suggested that the combination of Healthy and Dys groups was more effective than either group alone in uncovering the biggest separation between Healthy/Dys and Dia animals. Conversely, and despite the increased noise level in the fecal bile acid data, by combining both primary and secondary bile acids, PCA analysis in Figure 1b revealed that Healthy and Dia samples cluster separately and that Dys samples were spread evenly across Healthy and Dia groups. This was validated by subsequent Random Forest data analysis.

### 3.3. Combined Univariate and Multivariate Results Reveal Specific Plasma Lipids That Are Differential between Healthy, Dys, and Dia Animals

OPLS-DA and two sample t-test results were combined to identify lipids that best distinguished between Healthy, Dys, and Dia groups. Using a GAGE-based approach, we computed the statistical significance of the association between lipid classes and T2D status. Our plasma lipid panel included 394 lipids that were divided into 13 lipid classes, including 127 PCs, 93 TGs, and 30 SMs. However, only 9 of the 13 lipid classes had at least 1 significant lipid. A total of 369 lipids were detected in Healthy and Dys samples and organized into the 9 lipid classes (see Supplementary Table S1). Within each class, the total number and percentages of significantly different lipids between Healthy and Dys samples are summarized. Compared to Healthy sample extracts, only phosphatidylcholines (PCs), as a class, were significantly (GAGE *p*-value = 0.0129) elevated in Dys samples. The 42 lipids (21 lipids belonged to the PC class) that contributed most significantly to differentiating Healthy from Dys samples are listed in descending VIP order in Table S2. Conversely, from the 85 differential lipids (see Table S3 for full list), we observed 54 TGs to be predominantly different between the Dys and Dia groups. 

**Figure 2:**
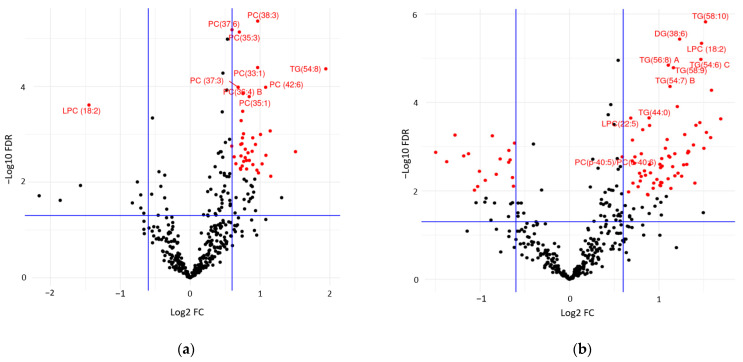
Volcano plot of −Log10(FDR) of adjusted *p*-value vs. Log2(FC) for all lipids following univariate and multivariate analyses. Red dots reveal the most significant features (i.e., the top ten annotated VIP-ranked lipids) for (**a**) Healthy vs. Dys and (**b**) Dys vs. Dia samples. The horizontal blue lines represent an FDR of 0.05, while the vertical blue lines indicate an FC of 1.5 and 1/1.5.

Following univariate and multivariate analyses of all lipids, a graphical summary of these results in the form of volcano plots revealed the top ten annotated VIP-ranked lipids, with the highest classification power for the Dys state (Figure 2a). These included LysoPC (18:2); long-chain PCs such as PC (37:6), PC (38:3), and PC (35:3); and TG (54:8). Conversely, and perhaps not unexpectedly, the top 10 VIP-ranked annotated lipids with the highest classification power for the Dia state (Figure 2b) mainly included TGs such as TG (58:10), TG (56:8), TG (58:9), TG (54:7), and TG (54:6). The numbers and percentages of significantly differential lipids and the class significance between Dys and Dia groups are summarized in Supplementary Table S4. The results revealed that the number of lipids that differentiate Dia from Dys is approximately twice the number of those that differentiate Dys from Healthy.

Using the VIP score rankings as input, we observed that PCs were significantly elevated in Dys animals compared to Healthy animals and that TGs were unchanged. Conversely, TGs were significantly elevated in Dia compared to Dys animals, but PCs were not (Table 2). These observations are consistent with a previously reported clinical lipidomics study of Finnish men [33], where PCs (as a class of lipids) were not significantly different between Dys and Dia patients. NHP TGs were not significantly different between Healthy and Dys animals, but rather, our results revealed that PCs were associated with pre-diabetes, followed by persistent elevated TG levels in Dys-to-Dia progressors. None of the remaining plasma lipid classes were significantly different between animal groups (Tables S1 and S4).

### 3.4. Strong Association between HbA1c and Plasma Long-Chain Polyunsaturated TGs

**Figure 3:**
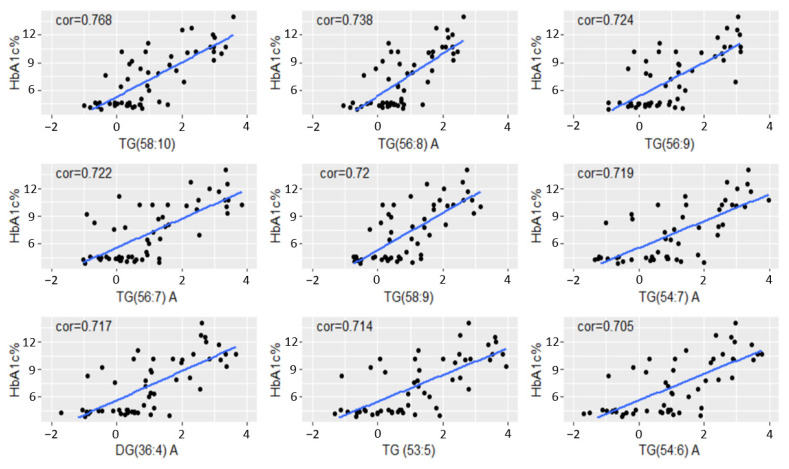
Pearson correlation plots (>0.70) of HbA1c% and various long-chain polyunsaturated DGs or TGs. The data was detrended by age, sex, and body weight. A and B denote structural stereoisomers of DG and TG. The blue lines represent fitted regression lines (cor) between HbA1c% and various TG lipids.

HbA1c levels are an important diagnostic tool for evaluating insulin resistance and disease severity [18]. Since palmitic acid has previously been positively correlated with HbA1c levels in T2D subjects [34], we sought to determine whether HbA1c levels, treated as a continuous variable, correlated with specific lipids on our list. We observed positive correlations (>0.70) between HbA1c and eight unsaturated, long-chain TGs and one DG (Figure 3) that were linearly associated with disease severity.

### 3.5. Fecal Secondary Bile Acids Can Distinguish Healthy from Dys/Dia Animals and Are Associated with T2D

Following propensity score matching that adjusted for BW, age, and sex, a weighted Random Forest classification algorithm was applied to fecal bile acid data to enable classification of different animal groups (Table 3). The classification accuracy between Healthy and Dys (AUROC = 0.67) was higher than that between Dys and Dia (AUROC = 0.59).

Moreover, when data from Dys and Dia animals were combined into a single Dys/Dia group vs. Healthy, we observed a higher classification accuracy (AUROC = 0.76) over either Dys or Dia alone. This result revealed that the fecal bile acids of Dys animals have a metabolic profile more similar to those of Dia animals than to those of healthy controls. Metabolites that contributed the most to predicting Healthy from Dys/Dia group association were ranked by their variable importance (VIMP) score, obtained from the Random Forest model.

**Figure 4:**
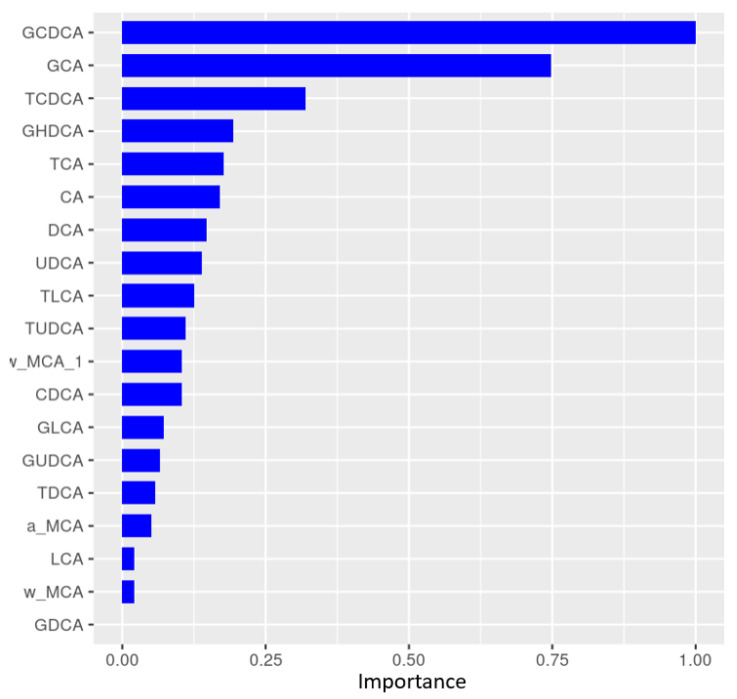
Variable Importance (VIMP) ranking of primary and secondary fecal bile acids following Random Forest analysis for disease classification of Dys/Dia or Healthy. All bile acids are expressed relative to GCDCA.

**Figure 5:**
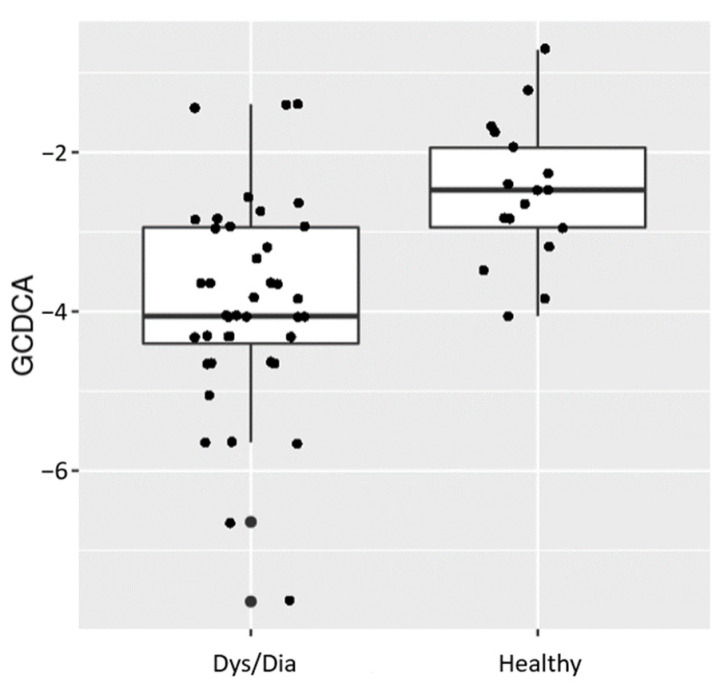
Boxplot of GCDCA abundances between two animal groups: Dys/Dia and Healthy.

The higher the importance score, the more influential this variable was when distinguishing Dys/Dia from Healthy. GCDCA and GCA were the most important fecal metabolites used to distinguish between different groups of animals (Figure 4). Univariate weighted t-tests revealed directionality and that GCDCA was most significantly reduced in diseased animals (FDR < 0.001). A boxplot of these results (Figure 5) shows the distribution of log-transformed GCDCA abundances across Healthy and diseased animals. Table S5 lists bile acids that were significantly reduced in Dys/Dia animals.

Through univariate AUROC analysis, we observed that for the fecal metabolomics data layer, the metabolites that were most predictive of Healthy and Non-Healthy (Dys/Dia) disease classification were several secondary bile acids: TCA, TUDCA, TCDCA, GCA, and GCDCA. They were all enriched in healthy subjects (see Table S6).

### 3.6. Metagenomics Results Reveal Several Bacterial Species to Be Associated with T2D

Analysis of the metagenomics data layer revealed that *Roseburia*, *Ruminococcus*, *Clostridium*, and *Streptococcus* were reduced in Dys/Dia animals (Table 4).

**Figure 6:**
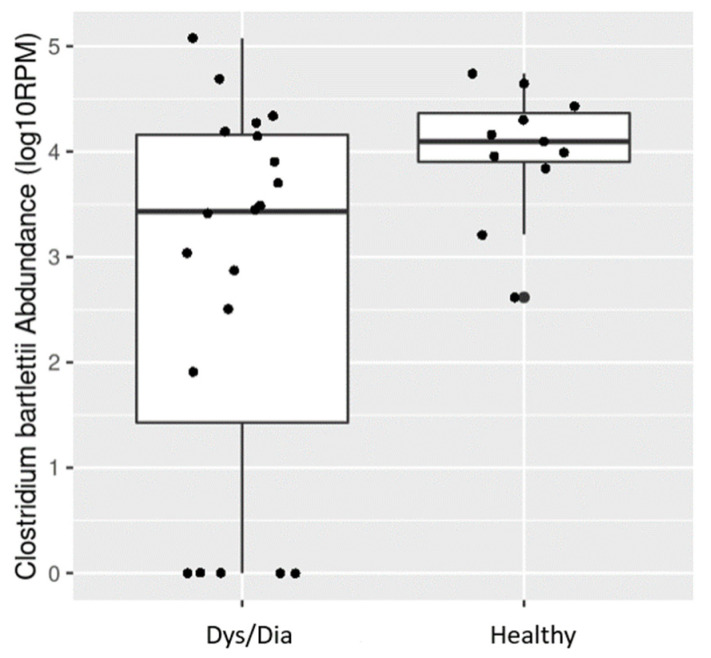
Boxplot of *Clostridium bartlettii* abundance between two animal groups: Dys/Dia and Healthy. The y axis is the abundance of *Clostridium bartlettii* species in the unit of reads per million on the log 10 scale (Log10 RPM).

Fecal bile acid concentrations were associated with the abundance of distinct bacterial taxonomic groups. Through inductive reasoning, we inferred that the abundance levels of these species were positively associated with the abundance levels of secondary bile acids and that they supported the classification of animals into the different groups created based on our metabolomics results. In an illustrative example, the boxplot of *Clostridium bartlettii* species’ taxonomic abundance between the two disease groups is provided in Figure 6.

## 4. Discussion

Like obesity and the development of insulin resistance, T2D progression in NHPs has a similar trajectory to that in human populations. Our principal aim was to determine if specific plasma and fecal metabolites/lipids from a colony of NHPs could be leveraged to classify which animals were healthy and which had developed pre-diabetes or diabetes. In addition to major anthropometric risk factors such as age, lifestyle, and diet, a prevailing feature of T2D development is increased insulin resistance, diet-induced obesity, and chronic accumulation of plasma lipids. The mechanisms by which specific lipid classes drive underlying disease progression, however, remain poorly understood. Several altered fatty acids and lipid classes have previously been reported to help improve the classification of human individuals at risk of developing diabetes. These include phospholipids (mainly PC and PE), TGs, DGs, cholesterol esters, ceramides, and sphingomyelins. Elevated plasma ceramides are known to correlate with dysmetabolic health and lower insulin sensitivity [35,36].

Both phospholipids and TGs exhibit some of the strongest associations with the risk of pre-diabetes and T2D development [10,37,38,39]. Conversely, decreased plasma lysophosphatidylcholine (LysoPC) levels are inversely or negatively associated with obesity, pre-diabetes, and T2D [40,41,42,43,44]. High serum levels of several sphingomyelins have also been shown to have a BMI-dependent association with metabolic syndrome and increased risk of T2D [45,46].

A large, population-based lipidomics profiling study of Finnish men adjusted their results for BMI and age so that any associations between metabolite/lipid levels and the development of T2D were not obscured by the imbalance of BW, age, and sex [47,48]. They identified and validated a signature of several plasma TGs, DGs, and phosphatidylcholines in participants progressing to T2D, including TG(17:1/18:1/18:2), PC(32:1), PC(34:2e), PC(36:1), and LysoPC(18:2). The authors used this signature to build a model for predicting early onset of prediabetes [33]. Interestingly, we observed several of the same PCs identified in the Finnish study participants to be significantly different in the NHPs, with VIP scores indicating that they contribute to differentiating between Healthy and Dys states (Table S1). However, in our study the lipids with the highest VIP scores greater than 2.0 were PC(35:3), PC(38:3), PC(37:6), PC(33:1), PC(35:1), PC (42:6), PC(36:4), PC (37:3), LPC (18:2), and TG(54:8). To what extent any of these differential lipids are present solely due to the metabolism of animal chow is unknown. Therefore, one must exercise care in over-interpreting the significance of detecting individual NHP plasma lipids. Even though acute changes to individual lipids and metabolites may not always be the same as in human cohorts, the molecular adaptations in metabolic pathways that lead to T2D should be the same.

In our study, elevated plasma TG levels were influenced by age. There was also a significant correlation between plasma TG and obesity (Table 1). We therefore applied detrending/propensity score matching for confounder adjustment. We uncovered distinct classes of lipids that were orthogonal to clinical attribute information. They helped with high-confidence disease state classification of the animals. Following data adjustment, we also observed that early onset of diabetes (Dys) was defined by significantly elevated PC levels compared to those of Healthy animals. A persistently elevated and significantly different TG lipid signature was observed in T2D progressors (Table 2). None of the remaining plasma lipid classes were significantly different between pairs of animal groups. Several TGs were positively correlated with HbA1c. For example, TG (58:10)—a polyunsaturated, longer-chain TG—was highly correlated with HbA1c (Figure 3), even after adjusting for BMI. Moreover, GAGE analysis confirmed that PCs were the most highly significant cluster of plasma lipids for differentiating between Healthy and Dys/Dia animals, whereas TGs differentiated the most between Dys and Dia animals.

Based on results of metagenomics and 16S rRNA gene sequencing, gut microbiome profiles have previously been associated with T2D outcomes in human populations [49,50]. By leveraging Mendelian Randomization (MR) analysis in a microbiome-wide association study, Sanna et al. was able to find a causal, host-driven genetic effect causing increased production levels of the SCFA, butyrate [51]. Connors et al. [52] have previously used paired gut microbiome and fecal–bile acid pool samples, obtained from a well-characterized cohort of pediatric CD patients, to identify a list of bacterial species belonging to butyrate-producing genera—*Roseburia*, *Ruminococcus*, *Clostridium*, and *Streptococcus*—that appear to play a key role in the conversion of primary to secondary bile acids.

The role of bile acid production and its association with T2D has been investigated by Sun et al. [53]. Their metagenomics and metabolomics results for newly diagnosed diabetic patients treated with metformin revealed altered bile acid metabolism of GUDCA, an FXR antagonist. A study of gut microbiota and intestinal FXR in mice revealed that GUDCA acted as an intestinal FXR antagonist that led to the improvement of metabolic endpoints in obese mice [54]. A study of the gut microbiome and its association with T2D involving individuals in urban Africa [49] suggested the possibility that modifying bacterial composition in the gut could be a viable strategy for improving glucose control.

We sought to confirm whether there were any potential interactions between the gut microbiome and the metabolites that might account for the three-group classification of animals. Our fecal metabolomics bile acids data had revealed that Dys animals had a more similar metabolic profile to Dia animals than to healthy controls (Section 3.5 of this paper). To be consistent with the fecal metabolomics analyses performed in this study, we also combined Dys and Dia animals into a single Dys/Dia group for the metagenomics analysis.

A few bacterial species (*Roseburia inulinivorans*, *Clostridium bartlettii*, *Ruminococcus obeum*, *Streptococcus pasteurianus*, and *Streptococcus lutetiensis)* were associated with T2D secondary bile acid abundance in non-healthy (i.e., Dys/Dia) animals (Table 4), and were found to have the same directionality of disease association as GCDCA, which was significantly enriched in non-healthy animals (Figure 5). This finding appears to suggest that a potential mechanism by which the NHP microbiome contributes to the development of T2D is through regulation of the conversion between some primary and secondary bile acids. Our VIMP results showed that glycoursodeoxycholic acid (GCDCA) and glycocholic acid (GCA) are the most important bile acids that could distinguish Heathy from Dys/Dia animals. Moreover, our results, although preliminary, suggest that future experiments on fecal bile acids and metagenomics could be extended to include a complex metabolic trait like diabetes (i.e., for correct classification of Dys/Dia animals as opposed to healthy ones).

Matching metabolomics and metagenomics data were available for only about one quarter of all the animals. Instead of performing correlative analysis directly between the metagenomics and the metabolomics data layers, we employed an inductive reasoning approach to identify positively associated bacteria–metabolite pairs through their indirect correlation with each disease group. More specifically, we first used all the available animal data for metagenomics and metabolomics to calculate the univariate descriptive statistics (i.e., AUROC and the associated 95% confidence intervals for classifying Healthy and Dys/Dia states), which were subsequently used to rank the metabolites and the bacteria species within their respective data layers. Next, the directionality of disease association for the top-ranking metabolites and bacterial genera/species were tabulated. The top-ranking disease-associated metabolites and bacteria species were considered a pair if both were enriched within a particular animal group. By inference, the corresponding bacteria and metabolite were believed to be positively interacting with each other by jointly driving the differences between Healthy and Dys/Dia animals. Such an ‘induced’ association between the bacteria taxa and the metabolites has been previously described in Crohn’s disease, where fecal bile acid data correlated with taxa from metagenome data [52].

In summary, several unsaturated, long-chain TGs were linearly associated with disease severity. Specifically, unsaturated TGs were highly activated in the plasma of Dia animals. In addition, our study found secondary bile acids to be downregulated in Dys/Dia animals. Combined with the results of our metagenomics analysis, the hypothesized interaction between microbiomes and the activity of bile acids in NHPs appears to be supported by our data.

## 5. Conclusions

Metabolites from plasma and feces offer independent information, suggesting different roles of fecal and plasma metabolites in understanding the etiology of T2D. Our results suggest prognostic value in performing both metabolomics and lipidomics analyses of plasma and feces. By integrating causal inference and machine learning techniques and applying them to our plasma metabolomics data, we were able to uncover distinct classes of metabolites, orthogonal to clinical attribute information, that helped with high-confidence disease state classification of the animals. Overall, based on our RF results, plasma metabolite profiles were more accurate in classifying an animal’s disease status at the time of collection, whereas fecal metabolite profiles, collected at roughly the same time as plasma, were a more accurate classifier of the potential for each animal to progress to a Dys/Dia state. As such, plasma and fecal metabolite profiles provide complementary information about the predisposition of a sedentary NHP animal model to develop T2D.

Hence, multi-omics approaches that integrate both host and microbial responses should facilitate a clearer overall understanding of how host–microbiome dynamics can be leveraged with ML and multivariate regression methods to pave the way for more precise differentiation of the progression from dysmetabolic to overtly diabetic states in NHP models of T2D. Ultimately, these approaches could translate into more informed treatment stratification of human volunteers that are more likely to respond to potential new treatments.

## Figures and Tables

**Table 1:** A summary table (prior to confounder adjustment) of key clinical and anthropometric measurements: body weight (BW); total triglycerides (TG); N = number of animals. Age, BW, glucose, HbA1c, TG, and LDL/HDL ratio are all expressed as the mean with standard deviation (SD).

Characteristic	Dia	Dys	Healthy
	N = 24	N = 16	N = 17
*Demographics*			
Female, Male	8, 16	5, 11	3, 14
Age	19.8 (4.6)	15.5 (3.2)	15.3 (5.8)
BW, kg	13.5 (4.0)	15.3 (3.6)	11.8 (3.5)
			
*Clinical assessment*			
Glucose, mg/dL	164.9 (40.1)	75.7 (31.7)	62.5 (5.2)
HbA1c, %	10.0 (2.2)	6.2 (1.6)	4.4 (0.3)
TG, mg/dL	360.5 (284.5)	157.1 (85.0)	62.0 (36.8)

**Table 2:** GAGE pathway analysis based on number (N) of lipids belonging to PC and TG lipid classes. * *p* < 0.05, *** *p* < 0.001.

Disease State	Class	N	*p*-Value
Healthy vs. Dys	PC	127	0.0142
	TG	93	0.6353 *
			
Dys vs. Dia	PC	127	0.7640
	TG	93	<0.001 ***

**Table 3:** Disease classification accuracy in fecal bile acids using Random Forest analysis.

Animal Phenotype	AUROC [95% CI]
Dys vs. Dia	0.59 [0.40, 0.79]
Healthy vs. Dys	0.67 [0.47, 0.87]
Healthy vs. Dys/Dia	0.76 [0.64, 0.89]

**Table 4:** Univariate descriptive statistics (i.e., AUROC and the associated 95% confidence intervals for predicting T2D status) reveal several bacterial species to be significantly reduced in Dys/Dia animals.

Species	AUROC [95% CI]	Dys/Dia vs. Healthy
*Roseburia inulinivorans*	0.31 [0.13, 0.50]	Down
*Clostridium bartlettii*	0.28 [0.10, 0.46]	Down
*Ruminococcus obeum*	0.28 [0.07, 0.49]	Down
*Streptococcus pasteurianus*	0.26 [0.09, 0.43]	Down
*Streptococcus lutetiensis*	0.26 [0.08, 0.44]	Down

## Data Availability

Datasets are available from the authors on written request. The West Coast Metabolomics Center (WCMC) facility, under the direction of Dr. Oliver Fiehn, generated the original raw LC/MS and LC/MS/MS data files. The Biocrates kit data was generated at Merck & Co. All data files which support the conclusions in our manuscript were analyzed at Merck & Co. Please submit a written request to the authors for the raw and/or processed data files, Supplementary Figures, and Tables.

## Supplementary Materials

The following supporting information can be downloaded at: https://www.mdpi.com/article/10.3390/metabo14030159/s1, Figure S1: Data pre-processing flow chart for untargeted panel; Table S1: The total number (N) of each class of detected lipids between Dys and Healthy samples; Table S2: Significant lipids by descending VIP order contributing the most to differentiating Healthy from Dys states. Structural stereoisomers are denoted by A, B and C; Table S3: Selected lipids (in descending VIP order) contributing to differentiating Dia from Dys. * FDR < 0.05; fc > 1.5|fc < (1/1.5) and vip > 1; Table S4: The number and percentage of significantly different lipids belonging to each class between Dys and Dia samples. FDR < 0.05; fc > 1.5|fc < (1/1.5) and vip > 1; Table S5: Conjugated bile acids are significantly reduced in Dys/Dia animals; Table S6: Fecal bile acids significantly changed in Dys/Dia animals. Univariate descriptive statistics (i.e., AUROC and the associated 95% confidence intervals for predicting T2D status).
