# Supplementary material for: Metabolomics and Lipidomics Analyses Aid Model Classification of Type 2 Diabetes in Non-Human Primates
Source: Metabolites. 2024 Mar 9;14(3):159. doi: 10.3390/metabo14030159 (PMC10972397; doi:10.3390/metabo14030159)
Supplement: Supplementary file 1 [file metabolites-14-00159-s001.zip › tables.pdf]

metabolites

MDPI

Table S1. The total number (N) of lipids in each class, the number and percentage of significantly different lipids between Dys and Healthy samples. *FDR <0.05; fc > 1.5 | fc<(1/1.5) and vip > 1

Class	N	Significant Lipids*	% within class	GAGE p-value
PC	127	21	16.5	0.0129
TG	93	7	7.5	0.8162
FA	29	4	13.8	0.4885
PE	27	3	11.1	0.2012
DG	12	3	25.0	0.4490
SM	30	1	3.3	0.9549
LPC	23	1	4.3	0.7732
GlcCer	3	1	33.3	0.2059
Cer	25	1	4.0	0.6166

Table S2. Significant lipids by descending VIP order contributing the most to differentiating Healthy from Dys states. Structural stereoisomers are denoted by A, B and C

Annotation	FC	FDR	VIP
PC(35:3)	1.6291	0.0010	2.3798
PC(38:3)	1.9543	0.0010	2.3796
PC(37:6)	1.5146	0.0010	2.2654
PC(33:1)	1.9531	0.0028	2.2511
PC(35:1)	1.7966	0.0054	2.1368
PC (42:6)	2.1149	0.0046	2.1325
PC(36:4) B	1.6885	0.0051	2.1221
PC (37:3)	1.6103	0.0046	2.0473
LPC (18:2)	0.3663	0.0074	2.0141
TG(54:8)	3.8418	0.0028	2.0138
PC(34:3) A	1.6837	0.0090	1.9501
PC(36:5) C	1.6584	0.0121	1.8901
PC (p-34:0) or PC (o-34:1)	2.2122	0.0187	1.8164
TG(58:5)	1.6978	0.0200	1.7891
PC (p-34:1) or PC (o-34:2) A	2.0098	0.0200	1.7673
PC(32:1)	1.6560	0.0297	1.7563
PC(36:1)	1.5741	0.0357	1.7519
PE (p-36:5) or PE (o-36:6)	1.6803	0.0214	1.7406
PC(34:4)	1.5108	0.0250	1.7343
TAG 55:4; TAG 18:1-18:2-19:1;	1.9117	0.0239	1.7332
PE (p-34:2) or PE (o-34:3)	1.8494	0.0279	1.7270
PC (38:3)	1.7275	0.0279	1.7046
FA (15:0) (pentadecylic acid)	1.8609	0.0214	1.7006
DG(36:2)	1.6525	0.0239	1.6966
PC(38:7)	1.6715	0.0239	1.6868
GlcCer (d40:1)	2.8442	0.0279	1.6376
DG(34:3)	1.7987	0.0325	1.6206
TG(58:9)	1.5475	0.0297	1.6145

PC (p-40:4) or PC (o-40:5)	1.8630	0.0362	1.5959
TG(54:4)	1.7486	0.0325	1.5877
TG(56:9)	1.7266	0.0297	1.5875
TG(54:2)	1.7510	0.0411	1.5852
FA (17:0) (margaric acid)	1.7754	0.0282	1.5743
PC (p-38:4) or PC (o-38:5) B	1.6465	0.0409	1.5719
PC(36:3) B	1.9695	0.0454	1.5549
PC (37:4)	1.7006	0.0331	1.5450
PE (p-38:4) or PE (o-38:5)	2.1205	0.0297	1.5284
DG(36:5)	2.0419	0.0357	1.5260
FA (13:0) (tridecylic acid)	1.6763	0.0379	1.5150
Cer(d42:2) B	2.2252	0.0482	1.4527
SM (d38:1)	1.9396	0.0419	1.4259
FA (28:0) (montanic acid)	1.6503	0.0411	1.4208

Table S3. Selected lipids (in descending VIP order) contributing to differentiating Dia from Dys. *FDR <0.05; fc > 1.5 | fc<(1/1.5) and vip > 1

Annotation	FC	FDR*	VIP
DG(38:6)	2.3479	0.0006	2.1354
PC(p-40:5)/PC(o-40:6)	1.5045	0.0152	1.9714
TG(58:10)	2.8705	0.0006	1.9499
TG(54:6) C	2.7686	0.0009	1.9051
LPC (18:2)	2.7829	0.0006	1.8916
TG(58:9)	2.2372	0.0009	1.8612
TG(56:8) A	2.1503	0.0009	1.8549
LPC(22:5)	1.6075	0.0062	1.8284
TG(54:7) B	2.1795	0.0022	1.7912
TG(44:0)	1.8526	0.0062	1.7474
TG(59:2)	2.1577	0.0152	1.7261
TG(57:1)	1.7444	0.0143	1.7194
PE (p-38:4) or PE (o-38:5)	0.3858	0.0167	1.7079
TAG 47:0; TAG 14:0-16:0-17:0;	1.6289	0.0104	1.6929
LPC(16:1)	0.6308	0.0157	1.6831
TG(62:2)	2.0503	0.0184	1.6714
TG(56:7) A	3.006	0.0023	1.6689
TG(55:1)	1.6543	0.0174	1.6589
FA (26:0) (cerotic acid)	0.6473	0.0353	1.6369
TG(64:2)	2.2618	0.0143	1.6211
TG(46:3) B	2.0152	0.0176	1.619
TAG 50:4; TAG 16:0-16:3-18:1;	2.6137	0.0121	1.6114
DG(38:5)	1.7635	0.0082	1.607
TG(54:5) B	1.8599	0.0069	1.6006
PC(32:1)	0.5496	0.0094	1.5971

TAG 58:7; TAG 18:0-18:1-22:6;	2.6645	0.0069	1.5917
SM (d33:1)	0.3542	0.0143	1.589
LPC(18:0)	1.6601	0.0152	1.5711
TG(52:4)	2.4427	0.0094	1.5614
DG(36:4) A	2.7465	0.0069	1.5404
PC(34:3) A	0.624	0.0168	1.5394
TG(46:0)	1.7424	0.0252	1.5257
TG(57:2)	1.8018	0.0247	1.5114
TG(49:3)	2.3189	0.0229	1.5081
PE (36:3)	0.4972	0.0216	1.508
FA (15:0) (pentadecylic acid)	0.5665	0.0239	1.5013
TG(56:1)	1.7879	0.0215	1.4993
FA (28:0) (montanic acid)	0.4401	0.0152	1.466
PC(36:4) B	0.6527	0.0118	1.4631
TG(54:7) A	3.2298	0.0062	1.4614
TG(60:1)	1.7024	0.0354	1.459
CE(16:1)	0.4568	0.0143	1.4573
TG(51:4)	2.9867	0.0099	1.4533
TG(62:3)	2.2629	0.0251	1.4401
TG(56:5) C	2.1027	0.0135	1.4372
LPC (20:0)	1.5805	0.0422	1.4356
TG(54:6) A	2.8895	0.009	1.4279
PE (p-36:5) or PE (o-36:6)	0.4114	0.0094	1.4112
TG(58:1)	1.7301	0.0299	1.409
TG(46:3) A	2.4822	0.0176	1.3936
TG(60:3)	2.1477	0.0277	1.3908
TG(62:4)	2.3849	0.0252	1.378
PC(33:1)	0.6247	0.0143	1.3747
FA (17:0) (margaric acid)	0.5839	0.0155	1.3741
TG(46:2)	2.0299	0.0315	1.3688
TG(56:9)	2.1311	0.0104	1.3613
TG(58:6)	2.5194	0.0143	1.3479
TG(64:3)	1.8311	0.0466	1.3411
SM (d34:1)	0.5203	0.0293	1.3385
DG(32:0)	1.7208	0.0229	1.336
TG(53:4)	2.5001	0.0143	1.3174
TG(51:5)	2.0261	0.0352	1.3111
TG(49:2)	2.6427	0.0315	1.3084
TG(50:5)	2.2246	0.0184	1.308
TG(52:5)	2.8341	0.0135	1.3015
TG(48:4) B	1.6353	0.0315	1.2996
FA (20:5) (eicosapentaenoic acid)	2.306	0.0045	1.2975
TG(56:6)	2.0436	0.0155	1.2874
TG(58:2)	1.9212	0.0315	1.2868

TG(48:4) A	1.8733	0.0229	1.2803
TG(58:3)	1.9127	0.0374	1.2782
TG(60:6)	2.1764	0.0152	1.2563
TG (53:5)	2.5106	0.0143	1.2529
PC(36:1)	0.6425	0.0263	1.2504
SM (d34:2)	0.4784	0.0393	1.2317
TG(58:4)	2.0458	0.0315	1.2017
FA (18:3) (linolenic acid)	1.8606	0.0176	1.2006
Cer(d42:2) B	0.4905	0.0354	1.1863
FA (18:2) (linoleic acid)	1.6259	0.0163	1.1825
TG(54:5) A	1.9687	0.0283	1.1767
TG(53:3)	2.0163	0.0347	1.1645
TG(60:2)	1.8369	0.0474	1.1587
TAG 52:6; TAG 16:1-18:2-18:3;	2.422	0.0176	1.142
TG(50:4)	2.1943	0.0374	1.1114
FA (22:6) (docosahexaenoic acid)	2.0256	0.019	1.0628

Table S4. The total number (N) of lipids in each class, the number and percentage of significantly different lipids between Dys and Dia samples. *FDR <0.05; fc > 1.5 | fc<(1/1.5) and vip > 1

Class	N	Significant lipids*	% within class	GAGE p-value
TG	93	54	58.1	4.2E-07
FA	29	8	27.6	0.4624
PC	127	6	4.7	0.9791
LPC	23	5	21.7	0.3552
DG	12	4	33.3	0.1887
SM	30	3	10.0	0.9944
PE	27	3	11.1	0.5856
Cer	25	1	4.0	0.9441
CE	10	1	10.0	0.8361

Table S5. Bile acids significantly reduced in Dys/Dia animals. Univariate weighted T-test result

Annotation	FC	FDR	VIMP (normalized)
GCDCA	0.2575	4.021E-07	1
GCA	0.2830	0.0043	0.75
TCDCA	0.3117	1.8927E-05	0.32
TCA	0.3670	0.0006	0.18
TLCA	0.4535	0.0143	0.13
TUDCA	0.3836	0.0007	0.11

Table S6. Feces bile acids significantly changed in Dys/Dia animals. Univariate descriptive statistics (i.e. AUROC and the associated 95% confidence intervals for predicting T2D status)

**Feces bile acids**	**AUROC and 95% CI**	**Dys/Dia vs. Healthy**
TCA	0.30 [0.16, 0.44]	Down
TUDCA	0.28 [0.15, 0.42]	Down
TCDCA	0.23 [0.11, 0.35]	Down
GCA	0.19 [0.07, 0.30]	Down
GCDCA	0.17 [0.06, 0.27]	Down
