# Supplementary figures and images for: Metabolomics and Lipidomics Analyses Aid Model Classification of Type 2 Diabetes in Non-Human Primates
Source: Metabolites. 2024 Mar 9;14(3):159. doi: 10.3390/metabo14030159 (PMC10972397; doi:10.3390/metabo14030159)

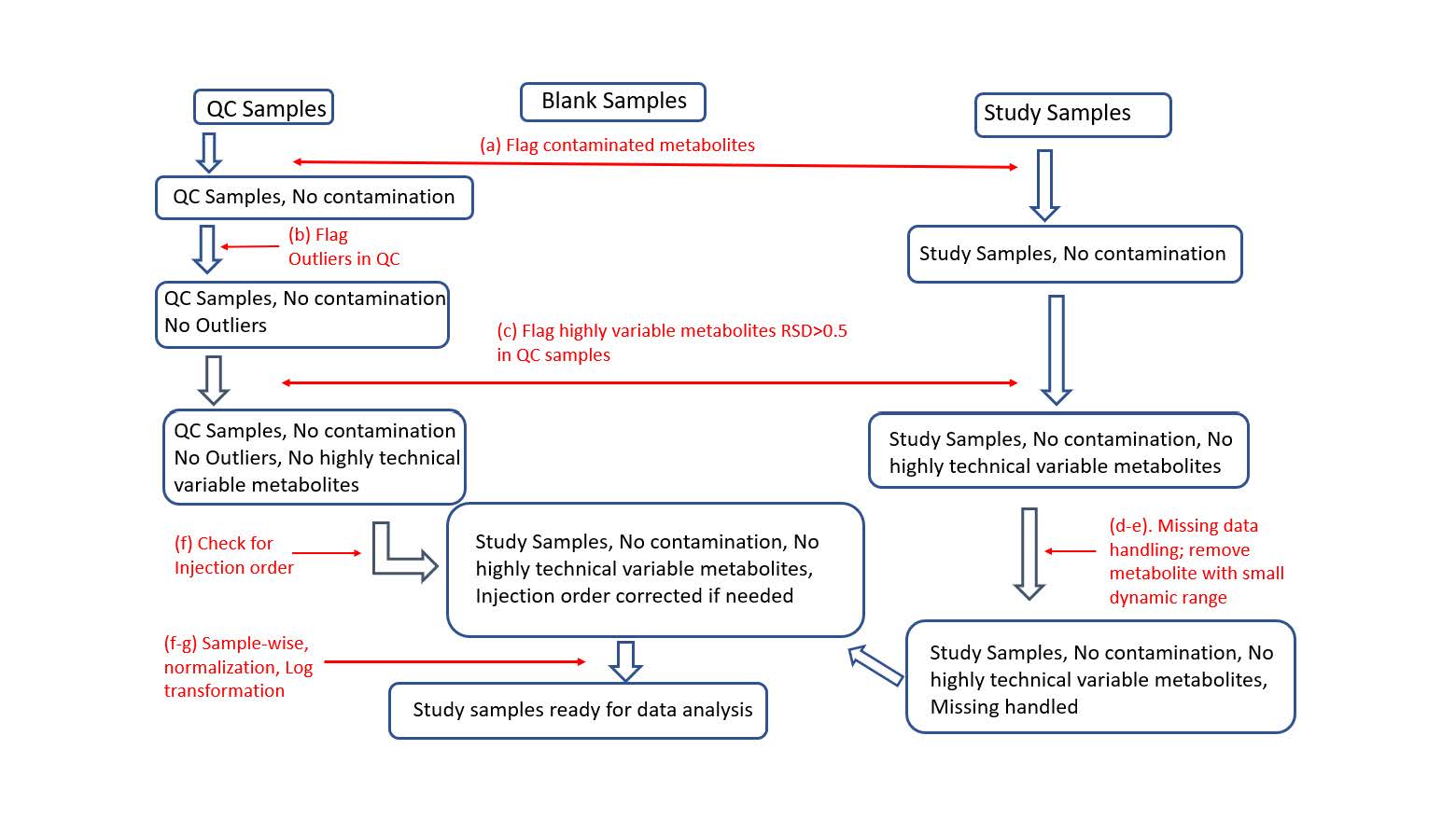

Supplement: Supplementary file 1 [file metabolites-14-00159-s001.zip › Figure S1.jpg]
